# Remodeling of the microbiota improves the environmental adaptability and disease resistance in Tibetan pigs

**DOI:** 10.3389/fmicb.2022.1055146

**Published:** 2022-11-30

**Authors:** Zhenyu Chang, Suxue Bo, Qingqing Xiao, Yu Wang, Xi Wu, Yuxuan He, Mujahid Iqbal, Yourong Ye, Peng Shang

**Affiliations:** ^1^College of Animal Science, Tibet Agriculture and Animal Husbandry University, Linzhi, China; ^2^The Provincial and Ministerial Co-founded Collaborative Innovation Center for R & D in Tibet Characteristic Agricultural and Animal Husbandry Resources, Linzhi, China; ^3^Key Laboratory of Clinical Veterinary Medicine in Tibet, Tibet Agriculture and Animal Husbandry University, Linzhi, China; ^4^Department of Pathology, Cholistan University of Veterinary and Animal Sciences (CUVAS), Bahawalpur, Pakistan

**Keywords:** Tibetan pig, growth stage, intestinal microbiota, 16S rRNA sequencing, Tibet

## Abstract

**Introduction:**

The establishment of intestinal microbiota and the maintenance of its equilibrium structure plays an important role in Tibetan pigs during different growth stages. Understanding the structure and function of the intestinal microbiota at different growth stages of Tibetan pigs can provide a theoretical basis for guiding nutritional regulation and feeding management in different stages.

**Methods:**

Fecal samples were collected from the Tibetan piglets at different growth stages, and the 16S rRNA was sequenced to analyze the changes of intestinal microbiota.

**Results:**

Alpha and Beta diversity indexes showed that the diversity of the intestinal microbiota did not change during the three growth stages, and the main components of intestinal microbiota were not significantly different. At the phylum level, Firmicutes and Bacteroidetes were dominant and abundant at different growth stages and were not restricted by age. At the genus level, Streptococcus, Lactobacillus, and Bifidobacterium were the most dominant in the TP10d and TP40d groups, Streptococcus was the most dominant in the TP100d group, followed by Treponema_2 and Lactobacillus. Fusobacteria, Gluconobacter, and Synergistetes were found to be specific genera of 10-day-old Tibetan piglets by LEfSe combined with LDA score. The change of diet made Tenericutes and Epsilonbacteraeota, which are closely related to digestive fiber, become specific bacteria at the age of 40 days. With the consumption of oxygen in the intestine, obligate anaerobes, such as Verrucomicrobia, Fibrobacter, and Planctomycetes, were the characteristic genera of 100 days. KEGG function prediction analysis showed that the intestinal microbiota function of Tibetan pigs changed dynamically with the growth and development of Tibetan piglets.

**Discussion:**

In conclusion, the structure and composition of the intestinal microbiota of Tibetan pigs are significantly different at different growth and development stages, which plays an important role in their immune performance.

## Introduction

Tibetan pig is a unique and geographically isolated pig breed living in the high-altitude area of the Qinghai–Tibet Plateau in China (Zhao et al., 2019). It is a typical highland miniature pig breed and is often used as an experimental material in the study of pig growth traits. Tibetan pigs are widely distributed in southeast Tibet, Sichuan Ganzi and Aba, Yunnan Diqing, and Gannan region. These pigs mainly live in the mountains, valleys, forests, and grassland. Their excellent features such as bearing high-altitude weather environment and disease resistance are closely related to the unique Tibetan pig intestinal flora (Yang et al., 2011; Shang et al., 2021). However, harsh natural conditions combined with extensive feeding and management also lead to a slow growth rate and low reproductive performance (Chen et al., 2014; Wang et al., 2017). For a long time, Tibetan pigs have been one of the main sources of meat for the Tibetan people. It is reported that these pigs are being raised in Tibet since the seventh century (Ma et al., 2019). Their population has experienced a lot of genetic differentiation, but Tibetan pigs of different populations can well adapt to the harsh plateau environment (Ai et al., 2014). For example, crude feeding tolerance is generally considered to be related to the ability of Tibetan pigs to digest fiber foods. As far as Tibetan pigs are concerned, digestive enzymes secreted by themselves cannot decompose fibrous substances, and can only rely on the function of microorganisms in the gut, especially in the cecum and colon.

Intestinal microbial flora composition of pig and human are similar, mostly including bacteria, archaea, eukaryotic organisms. There are about 1,000 kinds of intestinal microbes mainly anaerobic bacteria and facultative anaerobic bacteria. Anaerobic bacteria such as lactobacillus are accounted for more than 99% in the maintenance of physical health, improvement of immunity and nutrients absorption metabolism. However, aerobic bacteria and facultative anaerobic bacteria account only for about 1% (Kim and Isaacson, 2015; Donaldson et al., 2016; Xiao et al., 2017). There are significant differences in the intestinal microbiota of pigs at different stages. During the embryonic period, the intestinal tract is in a sterile state. During parturition, microorganisms, mainly *E. coli* and Staphylococcus, begin to appear under the influence of the maternal birth canal, feces, and the surrounding environment (Pluske, 2016). At different growth stages, the dominant bacteria in the intestinal tract of pigs are mainly Firmicutes and Bacteroides. The dominant bacteria in the intestinal tract of pigs are closely related to the regulation of autoimmunity. These dominant bacteria in the intestinal tract see a change with age and external environment accordingly (Isaacson and Kim, 2012).

The gastrointestinal microbiota of pigs is a heterogeneous ecosystem dominated by bacteria (Yang H. et al., 2017), and intestinal bacteria exert a significant impact on the host nutrition, physiology and immune processes in a variety of ways (Maltecca et al., 2020). With the emergence of low-cost and high-throughput sequencing technology, studies on animal intestinal microbiota have increased dramatically. Commercial pigs are expensive to feed, have a long growth cycle, and are difficult to deal with. Therefore, many studies mainly focus on the composition and diversity of intestinal microbiota in pigs at a certain stage, but there are few studies on the overall longitudinal changes of intestinal microbiota dynamics in Tibetan pigs at different growth stages (Armougom and Raoult, 2009; Crespo-Piazuelo et al., 2018; Bergamaschi et al., 2020). Therefore, studying the characteristics of intestinal microbiota at different growth stages of Tibetan pigs can play an important role in the healthy growth of Tibetan pigs, and can better reveal the composition and balance mechanism of intestinal microbiota at different growth stages, which is of great significance for the development of plateau animal husbandry.

With the development of microbiome, metabolomics, aseptic technology, and fecal microbiota transplantation (FMT) technology, the role of porcine intestinal microbiota in nutrient digestion, absorption and utilization has gradually become a hot topic. The dynamic balance of intestinal microbiota in pigs is an important prerequisite to ensure the normal digestion, absorption, and metabolism of nutrients. Therefore, this study aims to explore the characteristics of intestinal microbiota in Tibetan pigs at different growth stages, in order to provide a reference study of the interaction mechanism between intestinal microbiota and host and to provide a basis for using intestinal microbiota as a regulatory target to improve intestinal health and improve production performance of pigs. In this study, we collected fecal samples from Tibetan piglets at 10 days (piglet), 40 days (nursery period), and 100 days (finishing period), and 16S rRNA gene high-throughput sequencing method was used to explore the longitudinal changes of intestinal microbiota at different growth stages.

## Materials and methods

### Sample collection

The current study was conducted on the Tibetan Plateau, the highest distribution of grazing pig species in the world. The experimental samples were collected from the experimental base of Tibet Agriculture and Animal Husbandry College in Nyingchi City, Tibet Autonomous Region (average altitude 2,980 m, longitude 94.34°, latitude 29.67°). Fresh fecal samples were collected from 6 Tibetan pigs with similar body weight in each litter at three time points of birth day 10 (piglet), day 40 (nursery period), and day 100 (finishing period). There were 6 Tibetan piglets, 6 nursery pigs, and 6 finishing pigs (labeled as TP10d-1, TP10d-2, TP10d-3, TP10d-4, TP10d-5, TP10d-6, TP40d-1, TP40d-2, TP40d-3, TP40d-4, TP40d-5, TP40d-6, TP100d-1, TP100d-2, TP100d-3, TP100d-4, TP100d-5, TP100d-6). These pigs were fed in half indoor feeding way, feeding without antibiotic with fodder, raising management were in accordance with the conventional procedures. Insect repellent and vaccination were done regularly, all pigs were free to gather at the feed and drinking water points. Stool samples were collected on the same day after getting target age and were mixed with litter of piglets samples as a group, cryopreserved in tubes at −80°C.

### Total DNA extraction, PCR amplification, high throughput sequencing

Fecal genomic DNA extraction kit (Guangzhou Meiji Biotechnology Co., LTD., D3141) was used to extract the total DNA from fecal samples. The concentration and purity of DNA were detected by 1% agarose gel. The total amount of DNA was determined using a Nano Drop NC 2000 (Thermo Fisher Scientific). The V3–V4 region of 16S rRNA gene was selected as the target fragment for amplification. For PCR the pre-primer sequence used was: 341F (5′-CCTACGG GNGGCWGCAG-3′) and 806R was: (5′-GGACTACHVGGGTATCTAAT-3′). The samples were uniformly diluted to 20 ng μL^–1^ as a template for PCR amplification. Amplification system (25 μL) was: PCR-Mix 12 μL, upstream primer (10 μm) 1 μL, downstream primer (10 μm) 1 μL, DNA template 2 μL, DD H_2_O 9 μL. The amplification parameters were as follows: pre-denaturation at 98°C for 2 min, denaturation at 98°C for 15 s, annealing at 55°C for 30 s, extension at 72°C for 30 s, 30 cycles, and extension at 72°C for 5 min. PCR products from the same sample were mixed and recovered on 2% agarose gel. AxyPrep DNA Gel Extraction Kit (Axygen Biosciences, Union City, CA, USA) was used to purify the recovered products. The recovered products were detected by 2% agarose gel electrophoresis and quantified by Quantus™ Fluorometer (Promega, USA). Illumina Mi Seq PE250 sequencer was used for sequencing.

### Bioinformatics and statistical analysis

In order to obtain accurate and reliable results in subsequent bioinformatics analysis, paired-end clean reads were merged as raw tags using FLSAH (Magoč and Salzberg, 2011) (version 1.2.11) with a minimum overlap of 10 bp and mismatch error rates of 2%. Noisy sequences of raw tags were filtered by QIIME (Caporaso et al., 2010) (version 1.9.1) pipeline under specific filtering conditions (Bokulich et al., 2013) to obtain the high-quality clean tags. Clean tags were searched against the reference database^1^ to perform reference-based chimera checking using the UCHIME algorithm.^2^ All chimeric tags were removed and finally obtained effective tags were used for further analysis. The effective tags were clustered into operational taxonomic units (OTUs) of ≥ 97% similarity using UPARSE (Edgar, 2013) pipeline. The tag sequence with the highest abundance was selected as a representative sequence within each cluster. Between groups Venn analysis was performed in R project (version 3.4.1) to identify unique and common OTUs. The effective tags were clustered into OTUs of ≥ 97% similarity using UPARSE [4] pipeline. The tag sequence with the highest abundance was selected as a representative sequence within each cluster. Between groups Venn analysis was performed in R project (version 3.4.1) to identify unique and common OTUs. The representative sequences were classified into organisms by a naive Bayesian model using RDP classifier (Wang et al., 2007) (version 2.2) based on SILVA (Pruesse et al., 2007) Database,^3^ with the confidence threshold values ranged from 0.8 to 1. The abundance statistics of each taxonomy were visualized using Krona (Ondov et al., 2011) (version 2.6). Biomarker features in each group were screened by Metastats (White et al., 2009) (version 20090414) and LEfSe software (Segata et al., 2011) (version 1.0). Chao1, Simpson, and all other alpha diversity index were calculated in QIIME. OTU rarefaction curve and rank abundance curves were plotted in QIIME. Alpha index comparison between groups was calculated by Welch’s *t*-test and Wilcoxon rank test in R project. Alpha index comparison among groups was computed by Tukey’s HSD test and Kruskal-Wallis *H*-test in R project. Weighted and unweighted unifrac distance matrix were generated by QIIME. Multivariate statistical techniques including PCA (principal component analysis), PCoA (principal coordinates analysis) and NMDS (non-metric multi-dimensional scaling) of (Un) weighted unifrac distances were calculated and plotted in R project. Statistical analysis of Welch’s *t*-test, Wilcoxon rank test, Tukey’s HSD test, Kruskal-Wallis *H*-test, Adonis (also called Permanova) and Anosim test were calculated using R project.v. The KEGG pathway analysis of the OTUs was inferred using Tax4Fun (Aßhauer et al., 2015) (version 1.0).

In order to explore the differences of bacterial community structure and diversity in three different growth stages, IBM SPSS 21.0 software was used to analyze the variance of each data. If the variance was significant, Duncan’s method was used for multiple comparisons, and the results were expressed as mean ± standard deviation, *P* < 0.05 means significant difference, *P* > 0.05 indicates no significant difference.

## Results

### Sequence analysis

In the current study, 18 samples from three groups were used to conduct amplicon sequencing to investigate the changes in gut microbiota. Results indicated that a total of 2,098,941 (TP10 = 741,257, TP40 = 708,960, TP100 = 648,724) raw sequences were generated (Table 1). Furthermore, 1,756,695 (TP10 = 623,221, TP40 = 592,483, TP100 = 540,991) valid sequences were obtained after quality assessment, with an effective rate of over 80%. According to 97% nucleotide-sequence similarity, the effective sequences were clustered into 1,781 OTUs and 654 OTUs were in common. Moreover, the number of unique OTUs in the TP10, TP40, and TP100 groups were 384, 207, and 182, respectively. Notably, rarefaction curves and species rank curve of all samples showed a tendency to saturate, indicating sufficient sequencing evenness and richness.

**TABLE 1:** Quantitative statistics of tags and OTUs.

Samples name	Raw reads	Clean reads	Raw tags	Clean tags	Chimera	Effective tags	Effective ratio (%)	OTUs
TP40-1	125,780	125,681	124,108	123,296	18,320	104,976	83.46	1,644
TP40-2	125,197	125,091	123,632	122,970	18,363	104,607	83.55	1,568
TP40-3	129,850	129,726	128,063	127,361	18,699	108,662	83.68	1,524
TP40-4	117,609	117,482	116,084	115,272	16,278	98,994	84.17	1,381
TP40-5	115,966	115,839	114,410	113,822	17,423	96,399	83.13	1,422
TP40-6	94,558	94,474	93,342	92,961	14,116	78,845	83.38	1,402
TP100-1	96,350	96,263	95,089	94,616	14,612	80,004	83.03	1,171
TP100-2	76,337	76,283	75,270	74,952	10,903	64,049	83.9	1,203
TP100-3	114,888	114,778	113,447	112,769	17,182	95,587	83.2	1,622
TP100-4	116,116	116,013	114,499	113,713	16,959	96,754	83.33	1,392
TP100-5	123,126	123,035	121,522	120,787	18,626	102,161	82.97	1,620
TP100-6	121,907	121,825	120,413	119,582	17,146	102,436	84.03	1,463
TP10-1	131,700	131,605	129,911	129,284	19,352	109,932	83.47	1,476
TP10-2	134,758	134,668	133,054	132,312	20,231	112,081	83.17	1,530
TP10-3	120,261	120,189	118,720	117,858	16,431	101,427	84.34	1,141
TP10-4	128,071	127,968	126,198	125,622	18,784	106,838	83.42	1,598
TP10-5	109,240	109,137	107,768	107,222	15,332	91,890	84.12	1,312
TP10-6	117,227	117,121	115,330	114,819	13,766	101,053	86.2	1,393

### Analysis of microbial composition and structure

**FIGURE 1:**
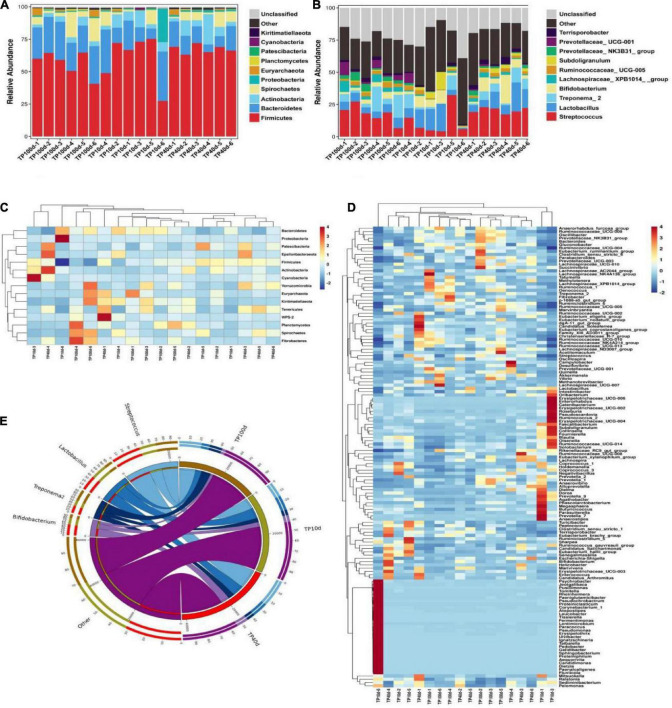
Relative abundance and heat map of intestinal microflora in Tibetan pig at phylum and genus levels at different growth stages. **(A,B)** Represent the community distribution at the phylum and genus level, respectively. **(C)** Heat maps of the top 17 common gates in different communities. **(D)** Heat maps of the top 96 common genera in different communities. Each color block in the heat map represents the relative abundance of a genus in the sample. Clustering can distinguish taxons with different abundance, and color gradient and similarity is reflecting the similarities and differences of multiple samples at different classification levels. The blue-red gradient shows the change of abundance from low to high. **(E)** The composition of microorganisms among horizontal species.

**FIGURE 2:**
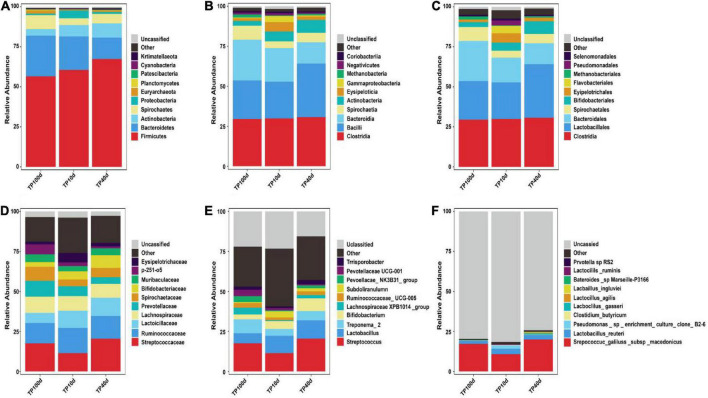
Relative abundance (Top10) of intestinal microflora in Tibetan pigs. At boundary **(A)**, phylum **(B)**, class **(C)**, order **(D)**, family **(E)**, and genus **(F)** levels at different growth stages.

At the phylum level, the Firmicutes (60.53, 67.37, and 56.51%) and Bacteroidetes (20.88, 13.32, and 25.45%) were abundantly present in TP10d, TP40d, and TP100d groups, regardless of age, which accounted for over 80% of the total taxonomic composition. Other phyla such as Planctomycetes (0.096, 0.23, and 0.52%), Patescibacteria (0.26, 0.33, and 0.15%), Cyanobacteria (0.33, 0.24, and 0.16%), and Kiritimatiellaeota (0.11, 0.15, and 0.28%) in TP10d, TP40d, and TP100d groups were identified in low abundances (Figures 1A–E). Among recognized genera, Streptococcus (11.44%, 20.47%), Lactobacillus (10.77%, 11.40%), and Bifidobacterium (4.93%, 7.96%) were the most predominant genus in the TP10d and TP40d groups, whereas the predominant genera observed in the TP100d group was Streptococcus (17.61%), followed by Treponema_2 (8.67%) and Lactobacillus (6.26%) (Figures 2A–F).

### Analysis of intestinal microflora diversity in Tibetan pigs at different growth stages

**FIGURE 3:**
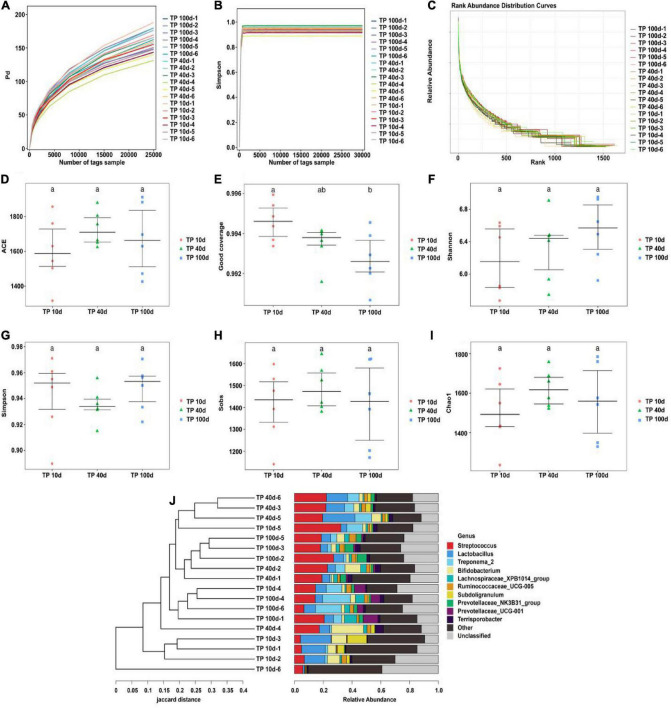
Microbial diversity in colon of Tibetan pig at different growth stages. **(A)** PD diversity index curve. **(B)** Simpson diversity index curve. **(C)** Rank abundance curve. **(D–I)** Alpha diversity index (Simpson, PD, Good’s coverage, Shannon, Chao1, and ACE). **(J)** UPGMA cluster tree. Each curve represents a sample.

The qualified sequences were aligned to evaluate multiple alpha-diversity indexes that could describe the diversity and abundance of the community. Good’s coverage estimations of each group varied from 99.06 to 99.59%, suggesting almost all bacterial phenotypes were identified. Statistical analysis of alpha diversity revealed that there were no obvious differences in the Chao1 (1503.31 ± 175.12 vs. 1622.74 ± 93.44 vs. 1557.03 ± 194.20), ACE (1601.99 ± 193.13 vs. 1729.90 ± 100.53 vs. 1669.86 ± 202.54), Simpson (0.941 ± 0.029 vs. 0.93 ± 0.013 vs. 0.94 ± 0.017), and Shannon (6.17 ± 0.43 vs. 6.32 ± 0.41 vs. 6.53 ± 0.39) indices between the TP10d, TP40d, and TP100d groups, suggesting that the diversity of gut microbiota did not change in Tibetan pigs at this stage. Furthermore, beta diversity analysis showed that the dots in the TP10d, TP40d, and TP100d groups were clustered together, indicating that there was no significant difference in the main components of gut microbiota among the three groups (Figures 3A–J).

### Analysis of microbial representation species of Tibetan pig at different growth stages

**FIGURE 4:**
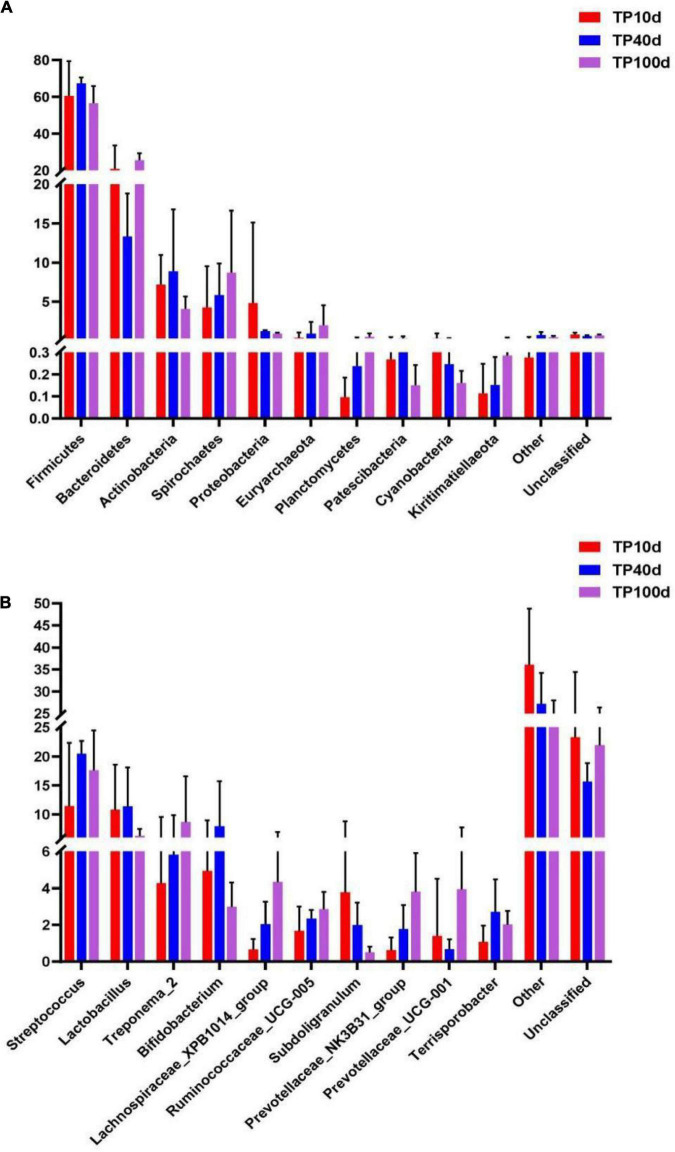
Comparison of community differences in intestinal microbial composition between Tibetan pigs. At phyla level **(A)** and genus level **(B)**. All data represent averages.

The previous analysis showed that there were great differences between Tibetan pig group and Yorkshire pig group at the gate level and genus level, so the microbial community composition of the two levels was analyzed, and the results are shown in Figure 4. As it can be observed in Figure 4A, the top 10 phyla are Firmicutes, Bacteroidetes, Euryarchaeota, Actinobacteria, Fusobacteria, Spirochaetes, Proteobacteria, Synergistetes, Patescibacteria, and Kiritimatiellaeota. The actinomycetes and spirochetes in the colon of Tibetan and Yorkshire pigs were the dominant communities, accounting for 81.15 and 76.26%, respectively. The relative abundance of actinomycetes and spirochetes in Tibetan pigs was significantly higher than that in Yorkshire pigs (*P* < 0.05). There were no significant differences in the relative abundance of other bacteria between Tibetan pigs and Yorkshire pigs (*P* > 0.05). It can be seen from Figure 4B that there are great differences in the composition of microflora at the genus level between Tibetan pigs and Yorkshire pigs, and the relative abundance of most microflora in Tibetan pig colon is higher than that of Yorkshire pig group. The relative abundance of Clostridium_sensu_stricto_1 (21.08%), Lactobacillus (10.44%), Sporobacillus (6.98%), Streptococcus (6.50%), and Ruminococcaceae_UCG-005 (3.36%) in the colon microbiota of Tibetan pigs was significantly higher than that of Yorkshire pigs (Clostridium_sensu_stricto_1 7.73%, Lactobacillus 1.83%, Bacillus 4.53%). Streptococcus 2.90% and Ruminococcaceae_UCG-005 2.36%) (*P* < 0.05).

**FIGURE 5:**
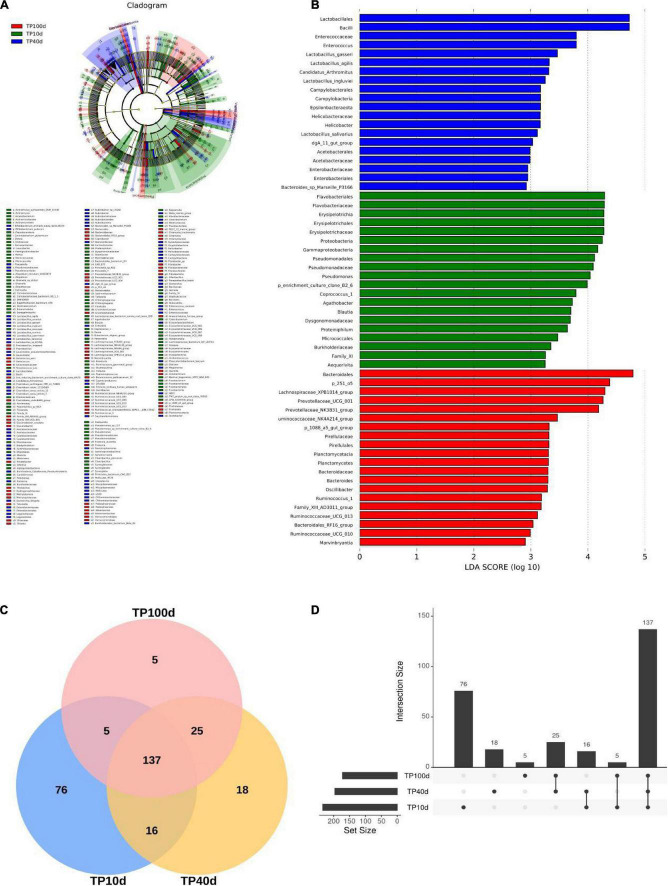
Comparison of differences in gut microbiota of pigs at different days of age. **(A)** and **(B)** are LEfSe analysis plots. **(C)** Venn plots of different age groups; **(D)** Figure shows the difference of gut microbiota between different days of age.

To further assess the changes in the gut microbiota of piglets at different ages, LEfSe combined with LDA scores were used for identifying the specific taxa associated with age change (Figure 5). At the phylum level, Verrucomicrobia, Fibrobacteres, and Planctomycetes were significantly more preponderant in the TP100d group than in the TP40d and TP10d groups, while the abundances of the Fusobacteria, Gluconobacter, and Synergistetes were dramatically increased in TP10d group in comparison with TP100d and TP40d groups. Additionally, the levels of Tenericutes and Epsilonbacteraeota tended to be higher in the TP40d group than TP100d and TP10d groups. We also observed that several genera such as Frateuria, Bacteroides, Prevotellaceae_UCG_004, Prevotellaceae_UCG_001, Oscillibacter, Chlamydia, Fructobacillus, Quinella, Prevotellaceae_NK3B31_group, Lachnospiraceae_UCG_001, Cetobacterium, Ruminococcus_1, Tatumella, Coprobacter, Lachnospiraceae_NK4B4_group, Family_XIII_UCG_001, Methylotenera, Ruminococcaceae_UCG_010, Anaerorhabdus_ furcosa_group, Sphaerochaeta, Thiobacillus, Ruminococcaceae_UCG_009, Gluconobacter, Oenococcus, Family_XIII_AD3011_group, p_1088_a5_gut_group, Lachnospiraceae_NK4A136_group, Fibrobacter, Ruminococcaceae_UCG_013, Akkermansia, Marvinbryantia, and Lachnospiraceae_XPB1014_group were the most dominant bacteria in the TP100d group as compared to TP10d and TP40d groups, whereas the proportions of Geobacillus, Escherichia_Shigella, Bradyrhizobium, Marivita, Megamonas, Marivivens, Acetobacter, Prauserella, LD29, Candidatus_Arthromitus, Helicobacter, Staphylococcus, Clostridium_sensu_stricto_15, NS3a_marine_group, Rubrobacter, Ureaplasma, Enterococcus, Cloacibacterium, Saccharofermentans, Erysipelatoclostridium, Caproiciproducens, Leuconostoc, dgA_11_gut_group, Eubacterium_eligens_group, Stenotrophomonas, DTU089, Ralstonia, Alteribacillus, Clostridium_sensu_stricto_7, Paraburkholderia, and CHKCI001 were significantly higher in the TP40d group than in the other two groups. Moreover, the TP10d group was significantly found enriched in Pasteurella, Rothia, Olsenella, Coprococcus_1, Fluviicola, Holdemanella, Globicatella, Candidimonas, Pseudomonas, Parvimonas, Dorea, Gemella, Lachnospiraceae_FCS020_group, Dietzia, Agathobacter, Erysipelotrichaceae_UCG_002, Erysipelotrichaceae_UCG_007, Erysipelotrichaceae_UCG_006, Erysipelotrichaceae_UCG_009, CPla_4_termite_group, Eggerthellaceae.DNF00809, Tissierella, Ruminococcus_gauvreauii_group, Fermentimonas, Roseburia, CAG_873, Actinomyces, Prevotella_7, Blautia, Dialister, Proteiniphilum, Taibaiella, Sediminibacterium, Denitrobacterium, Arcanobacterium, Leucobacter, Aequorivita, Ruminococcaceae_UCG_003, Pelomonas, Synergistia, Cloacibacillus, Atopobium, Peptostreptococcus, Shuttleworthia, Paeniglutamicibacter, Sharpea, Fusobacterium, Odoribacter, Enterorhabdus, Pseudoscardovia, Proteiniclasticum, Senegalimassilia, Acidaminococcus, Filifactor, Catenibacterium, and Collinsella compared with TP100d and TP40d group.

### Prediction of microbial ecological function of Tibetan pigs at different growth stages

**FIGURE 6:**
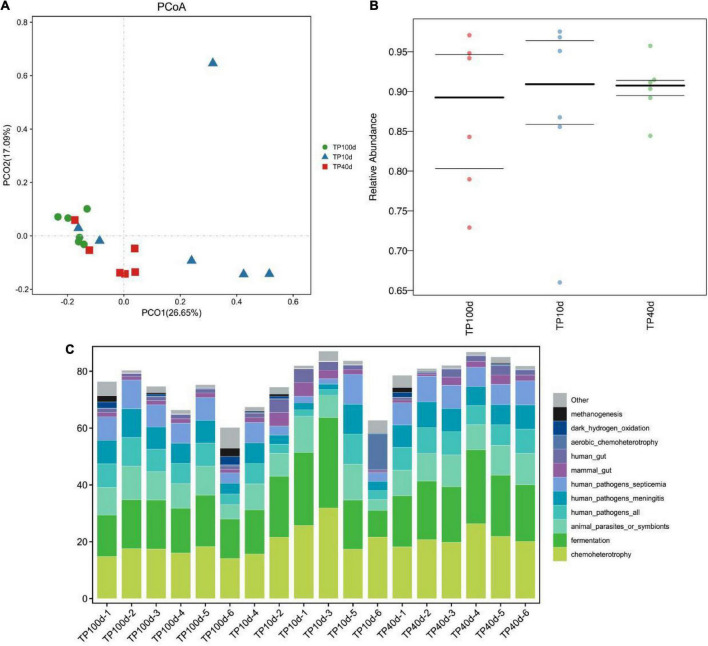
Prediction of intestinal microbial ecological function of Tibetan pig at different growth stages. **(A,B)** PCoA analysis of group TP10d, TP40d, and group TP100d at the genus level. Each point represents a sample. The distance between the two points indicates the difference in fecal microbiota. (CmurF) TP10d, TP40d, and TP100d intestinal microbial ecological function prediction **(C)**.

Principal coordinate analysis was performed to analyze the differences of intestinal flora at different growth stages. At the phylum level, the intestinal microbiota structure of suckling and nursery piglets slightly crossed but could be completely separated from that of newborn piglets (Figure 6A). At the genus level, piglets were able to fully separate their gut organisms at the three stages (Figure 6B).

In conclusion, the succession of intestinal microbiota changed significantly with increase in the age of pig. After using PICRUSt to predict the function of microorganisms, it was found that the function of intestinal microbiota changed dynamically with the growth and development of Tibetan pigs, and the microbial communities of the three groups of Tibetan pigs were mainly related to metabolism, disease function, cell transformation, genetic information processing, and environmental information processing, etc. The abundance of microbial communities for infectious diseases and immune diseases was the highest on the 10th day, and then showed a downward trend. Opposite pathways such as immune system, transcription, cellular community—Prokaryotes, transport and catabolism, and neurodegenerative diseases showed an increasing trend and reached the highest abundance at 100 days (Figure 6C and Table 2).

**TABLE 2:** Functional prediction of three groups of Tibetan pigs at different growth stages.

Level_1	Level_2	TP40d	TP100d	TP10d
M	Carbohydrate metabolism	260258.26	235265.96	265604.65
M	Amino acid metabolism	226694.69	213477.11	237281.99
M	Metabolism of cofactors and vitamins	214941.12	212421.52	221398.83
M	Metabolism of terpenoids and polyketides	162783.19	156734.83	172590.53
M	Metabolism of other amino acids	130978.09	117769.43	135952.77
M	Lipid metabolism	115140.38	94428.19	117474.1
M	Energy metabolism	96341.41	93074.48	101534.38
M	Xenobiotics biodegradation and metabolism	68869.13	57360.06	79682.61
M	Glycan biosynthesis and metabolism	56872.72	62626.92	61897.43
M	Biosynthesis of other secondary metabolites	41830.91	39899.42	41342.17
M	Nucleotide metabolism	40132.12	37772.72	40656.26
GIP	Replication and repair	118594.29	110072.92	121111.32
GIP	Translation	67531.76	63224.36	67091.48
GIP	Folding, sorting, and degradation	56622.48	53839.83	59120.81
GIP	Transcription	17936.21	18581.63	19640.47
CP	Cell motility	37998.58	41324.13	40583.28
CP	Cell growth and death	28246.43	27082.34	29229.23
CP	Transport and catabolism	4071.36	4109.7	4538.6
CP	Cellular community—prokaryotes	3155.53	3228.19	3518.75
EIP	Membrane transport	41220.89	34078.72	43818.69
EIP	Signal transduction	6546.61	6040.1	7256.9
EIP	Signaling molecules and interaction	0.32	0.11	0.63
OS	Environmental adaptation	3435.12	3466.01	3764.5
OS	Endocrine system	1832.36	1757.39	1840.6
OS	Immune system	898.55	1079.07	1126.72
OS	Digestive system	498.84	788.5	584.71
OS	Excretory system	0.02	0.01	0.26
HD	Infectious diseases	5316.17	4497.89	4761.07
HD	Neurodegenerative diseases	219.83	503.03	715.67
HD	Cardiovascular diseases	0.27	0	6.34
HD	Immune diseases	0.09	0.04	0.08

M, Metabolism; GIP, Genetic Information Processing; CP, Cellular Processes; EIP, Environmental Information Processing; OS, Organismal Systems; HD, Human Diseases.

## Discussion

Tibetan pigs are the main pig species on the Qinghai–Tibet Plateau, distributed in mountains, valleys, forests, and grasslands with an altitude of 2,900–4,100 m (Zhang et al., 2015). Age and microecological space of the host dynamically change under the influence of dietary composition, nutrient level, and environmental factors. Age, intake of solid feed, and weaning were the main driving forces of succession and establishment of intestinal microbial population in piglets (10 days). The stable intestinal microbiota was not established in piglets at early weaning. The introduction of solid diet and environmental changes disrupted the balance of intestinal microbiota in piglets, resulting in the deterioration of their health status and growth performance. The growth process of nursery pigs (40 days) accompanied by a series of adverse factors, such as house transfer, feeding mode, and feed change leads to the disorder of intestinal flora of nursery pigs. Diet was the dominant factor (57%) affecting the number and composition of intestinal microorganisms in finishing pigs (100 days), which was related to the health level and growth performance of finishing pigs. There are few studies on the longitudinal changes of intestinal microbiota in Tibetan pigs at different growth stages. Understanding the structure and function of intestinal microbiota in Tibetan pigs at different growth stages can provide a theoretical basis for guiding nutritional regulation and feeding management at different stages. With the increasing demand, Tibetan pig farming is gradually developing to a larger scale (Zhang et al., 2017). The gastrointestinal tract of pig has diverse and complex microbial communities. However, the composition of gut microbiota is not immutable, and its composition and ecological succession are determined by many complex internal and external factors. The gastrointestinal microbiome of pigs contains thousands of different microbial species, such as Firmicutes, Bacteroidetes, and Proteobacteria (Holman et al., 2017). The gastrointestinal tract of pigs begins to be colonized by microorganisms shortly after birth and gradually becomes stable over the time (Faith et al., 2013). The changes of intestinal microbiota structure can affect the health status and growth performance of pigs. Metagenomic analysis of fecal microbiota of piglets with diarrhea by Yang Q. et al. (2017) showed that diarrhea was associated with increased relative abundance of Prevotella, Sudella, Campylobacter, and Fusobacteriaceae bacteria. Therefore, it is important to understand the changes in intestinal microbiota during pig growth and development. Liu et al. (2019) found in their study that the intestinal microbes of piglets changed a lot before and after weaning. Bacteroides had the highest content in the intestinal tract within 1 week of birth. After weaning, this dominant position was replaced by Prevotella, which was closely related to fiber digestion. The finishing period occupies a large proportion in the whole breeding cycle of pigs. Although the composition and structure of intestinal microorganisms in pigs in the finishing period remain relatively stable, they are still in a state of dynamic change. With the progress of the finishing period, the level of Firmicutes in pig manure increases while the level of Bacteroides decreases (Ban-Tokuda et al., 2017). It is well known that diversity can improve the stability and function of gut microbiota, and in particular, gut microbiota diversity has been considered as a novel biomarker of health and metabolic activity (Clarke et al., 2014). At present, the gut microbiome of pigs at a certain growth stage is well understood, but still there is a lack of comprehensive longitudinal studies on the dynamics of gut microbiome in Tibetan pigs at different growth stages. Therefore, we collected fecal samples of Tibetan pig at three growth stages, 10, 40, and 100 days in order to observe gut microbes and its influencing factors for the sake of adopting new strategies to regulate the intestinal microbiome, thereby enhancing the Tibetan pig intestinal health and growth performance.

The results of this study showed that there was no significant change in the diversity of intestinal microbiota of Tibetan pigs at three growth stages of Tibetan piglets, nursery pigs, and finishing pigs. According to the Alpha diversity of intestinal microbiota in Tibetan pigs at three developmental stages, the intestinal microbiota diversity of piglets increased significantly from birth to 10 days, and reached the highest level at the time of conservation. Mi et al. (2019) found that the Alpha diversity of gut microbiota in miniature pigs increased dramatically with the age, increasing to about 20 weeks and then fluctuating slightly throughout life. Similar diversity results have been found in human infant gut microbiota studies (Chernikova et al., 2018). This indicates that the development of intestinal microbiota of newborn piglets is basically the same as that of human beings. When exposed to various bacteria in the environment, the number and diversity of intestinal microbiota will increase rapidly, which is also the key to the development of intestinal microbiota. The research results of Backhed et al. (2005) and Leamy et al. (2014) showed that intestinal microbes have a wide range of functions and are involved in almost all life processes, including resistance against potential pathogens, absorption of different kinds of nutrients, regulation of host immune system, fat deposition traits, chronic diseases, etc. Gut microbiota contributes greatly to the host health and nutrient digestion (Coleman et al., 2018; Kumar et al., 2018; Zitvogel et al., 2018; Lei et al., 2021). The results of PCoA analysis also showed that the composition of intestinal microbiota was different at different time periods from 10 to 100 days of birth, indicating that the structure and composition of intestinal microbiota may change greatly with the passage of time.

At the genus level, Ruminococcus appeared during lactation and reached its highest abundance during the finishing period. Ruminococcus is the main producer of butyric acid in the intestine, which can provide about 70% of the energy for intestinal epithelial cells (Serpa et al., 2010). Butyrate, a type of short-chain fatty acid, has been shown to be beneficial for intestinal development and maintenance of intestinal health in mammals and has immune defense functions (Ratajczak et al., 2019). The main bacteria producing butyrate are anaerobic bacteria, and the low oxygen concentration in the intestine creates a favorable environment for anaerobic bacteria. At the same time, butyrate absorbed and metabolized by the epithelium can consume oxygen in the intestine, thereby stabilizing hypoxia-inducible factor (HIF, a transcription factor that orchestrates barrier protection) and protect the barrier function of the epithelium (Kelly and Colgan, 2016). In addition, this study found that Lactobacillus was present in all three time periods. However, its abundance reached the highest in the fattening period. This is maybe due to change in feed, from breastfeeding to fattening period, the piglets feed change from milk to difficult feed digestion and absorption of solid particles. So, they were in need of more lactobacillus. This also illustrates the lactic acid bacteria help in maintaining the stability of the intestinal bacteria of piglets and increase the beneficial effects of piglet adaptability to the environment.

In this study, LEfSe combined with LDA score method was used to analyze and identify the specific microorganisms of intestinal flora of different age pigs at the phyla level. It was found that the intestinal specific bacteria at 10 days were mainly Fusobacteria, Gluconobacter, and Synergistetes. The pattern of nutrient intake has a big impact on the development of gut microbiota. For example, when piglets switch from breast feeding to a solid diet, the abundance of microbes associated with digestive fiber in their gut microbiota changes significantly. In this study, it was found that Tenericutes and Epsilonbacteraeota were specific bacteria for the nursing pigs (TP40d), and Verrucomicrobia, microbacillus, and Planctomycetes were specific bacteria for the finishing pigs (TP100d). Wang et al. (2019) found that the abundance of Prevotella was low in the lactation period and significantly increased after weaning, and continued to increase in the nursery and growth period, and got decreased in the fattening period. Prevotella is a Gram-negative anaerobe of the Bacteroidete phylum, which can ferment dietary fiber to produce short-chain fatty acids (Franke and Deppenmeier, 2018). The intestinal microbiota is closely related to the digestive function and dietary pattern of the host, which together exert an important influence on the growth and development of the host.

During growth and development, the development of mammalian gut microbiota is affected by breed, age, and diet, and reaches stability at maturity. The results of this study showed that Firmicutes (60.53, 67.37, and 56.51%) and Bacteroides (20.88, 13.32, and 25.45%) were the dominant phyla of intestinal flora in Tibetan pigs during the three growth stages. Pajarillo et al. (2014) explored the similarities and differences of fecal microbial communities of Duroc, Landrace, and Yorkshire pig by 16S rRNA gene sequencing, and found that at the phylum level, most sequences were classified into Firmicutes and Bacteroides regardless of pig breeds. But the abundance of gut microbiota changes during pig development. At the phylum level, Firmicutes and Bacteroidetes showed an increasing trend at different growth stages.

In this study, KEGG functional prediction analysis showed that the function of intestinal microbiota changed dynamically with the growth and development of piglets, among which the abundance of metabolic pathways such as cancer, neurodeformation disease and drug resistance was the highest at 0 day, and then decreased. This indicates that the gastrointestinal tract of piglets is immature at birth, and the intestinal microbiota is not fully developed, and the diversity is low, which is easy to be invaded by pathogens. On the contrary, pathways of immune system, biosynthesis, replication and repair of other secondary metabolites increased with the growth and development of piglets, and the highest abundance was observed at 40 days. Mi et al. (2019) also found that the relative abundance of secondary metabolism such as terpenoids and polyketides increased significantly with increasing age. This indicates that with the growth and development, the diversity of intestinal flora increases, and the abundance of microorganisms related to nutrient digestion increases, which promotes the metabolism and absorption of nutrients and maintains the health and normal development of the body.

At present, there are few studies on the effects of vitamin and mineral metabolism in Tibetan pigs. Future studies can also strengthen the understanding mechanism of Tibetan pig gut microbes’ interaction with the host nutrition, liver and brain gut shaft axis, to find the effective gut microbes control targets, and can help in proving the regulatory mechanism of Tibetan pig nutrition metabolism.

## Conclusion

In this study, Tibetan pigs in Nyingchi City, Tibet Autonomous Region were selected as subjects to analyze the diversity and functional changes of intestinal microbiota in three different growth stages. It was found that there was no significant change in the diversity of intestinal microbiota in Tibetan piglets. The relative abundance of specific gut microorganisms changes dynamically with the age. For example, at the genus level, aerobic and facultative anaerobic bacteria first appear in the gut of newborn piglets, and then are replaced by obligate anaerobic bacteria. With the increase in age and the development of gut microbiota, the function of gut microbiota also changes significantly. This study expands our understanding of the dynamic migration of intestinal microbiota in Tibetan pigs at different growth stages and provides a theoretical reference for studying the changes of intestinal microbiota colonization and succession in piglets.

## Data availability statement

The datasets presented in this study can be found in online repositories. The names of the repository/repositories and accession number(s) can be found below: NCBI—PRJNA894839.

## Author contributions

PS: study conception and design. ZC, SB, QX, YW, XW, YH, and YY: experimentation and data analysis. ZC, SB, QX, YW, XW, and YH: contribution toward reagents, materials, and analysis tools. PS, QX, SB, and ZC: writing and revising of the manuscript. All authors contributed to the article and approved the submitted version.

## Footnotes

1
http://drive5.com/uchime/uchime_download.html

2
http://www.drive5.com/usearch/manual/uchime_algo.html

3
https://www.arb-silva.de/
